# Response: Commentary: Greater Emotional Gain from Giving in Older Adults: Age-Related Positivity Bias in Charitable Giving

**DOI:** 10.3389/fpsyg.2016.01887

**Published:** 2016-11-30

**Authors:** Pär Bjälkebring, Daniel Västfjäll, Stephan Dickert, Paul Slovic

**Affiliations:** ^1^Department of Psychology, University of GothenburgGothenburg, Sweden; ^2^Department of Behavioural Sciences and Learning, Linköping UniversityLinköping, Sweden; ^3^Decision Research, University of OregonEugene, OR, USA; ^4^School of Business and Management, Queen Mary University of LondonLondon, UK

**Keywords:** charitable giving age, emotion, motivation, decision making

We thank Hargis and Oppenheimer (2016) for their interesting commentary to our article (Bjälkebring et al., 2016). Age-related changes in decision making are indeed a relatively unexplored phenomenon especially when it comes to more specific decision situations such as prosocial acts.

Prosocial behaviors are distinct in the way that they feature both positive and negative emotions, moral considerations as well as other highly cognitive determinants (i.e., calculations of utility and efficacy). When it comes to the influence of age related cognitive decline on decision making earlier studies (i.e., Salthouse, 1996) most probably have overestimated this decline due to cohort differences (i.e., The Flynn Effect; Rönnlund and Nilsson, 2009) and newer longitudinal studies (i.e., Rönnlund et al., 2005; Gerstorf et al., 2011) suggest that the cognitive decline associated with normal aging does not have much functional impact until the last decade in a person's life, this is especially evident in time to death models (Thorvaldsson et al., 2008). In addition, “cognitive decline” might be too broad of a term to describe what happens during the life-span, results from the Baltimore Longitudinal Study of Aging suggest that longitudinal cognitive aging of executive and memory functions is not a uniform process but a heterogeneous one (Goh et al., 2012). However, recent research has shifted from investigating age-related cognitive decline to investigating age-related cognitive changes, for example the positivity bias seems to be connected not to cognitive decline (i.e., degradation) but to cognitive changes (i.e., motivation; Kalenzaga et al., 2016). Additionally, evidence exists that there are tangible cognitive processes that differ between older and younger adults, for example the use of gist and verbatim memory processes (Flores et al., 2016). Largely, emotional maturity processes as well as life experience seem to improve decision making throughout the lifespan and research suggest that people peak as decision makers in the second half of the lifespan (Strough et al., 2015). Interestingly, current research into risk taking has suggested that changes in risk taking could be consistent with age-related dopaminergic decline (Rutledge et al., 2016). That being said, people continue to make decisions late in life, one example being a person's final will and testament, a major consideration regarding prosocial giving. Hence, a life-span view of age related biological, cognitive, motivational, and emotional change and decline is needed. When it comes to prosocial decisions specifically, not much research have been conducted related to age related changes. However, there are some age-related differences that could be expected based on current theories and findings. One of these is the compassion fade, meaning that more emotions are elicited toward one victim compared to many (Västfjäll et al., 2014). Additionally, young and old could possibly differ when it comes to moral considerations, such as the valuation of lives (Dickert et al., 2012).

We agree with Hargis and Oppenheimer (2016) that the positivity bias is not the only process related to affect or the processing of affect that could possibly influence age differences in prosocial decision making. First, an aspect we do not discuss is the age-related preference for different emotions. Based on Russell's (2003) notion that preference for affect can change when arousal changes, as well as previous research on age differences in arousal preference (Kessler and Staudinger, 2009; Mogilner et al., 2011; English and Carstensen, 2014). It is expected that older adults prefer low arousal emotions when compared to high arousal emotions. In line with this older adults rated their happiness lower when it was framed as high in arousal (ecstatic), and higher when it is was framed as low in arousal (satisfied; Bjalkebring et al., 2015). Secondly, older and younger adults seem to differ in the way they regulate their emotions. As an example adults use reappraisal more often than younger adults (Gross and John, 2003). When it comes to decision making one of the most important emotions is regret. Research suggests that older and younger adults may differ in their experience, anticipation, and regulation of regret partly because the opportunity to overcome regret declines with age (Peters et al., 2011). This is illustrated by the research of (Wrosch and Heckhausen, 2002) as well as (Wrosch et al., 2005). In their studies, participants were asked to report activities that they regretted not having pursued during their lives and to indicate the amount of personal control they had on the situation at the time. Both the experience and regulation of regret differed between younger and older adults. When younger adults reported that they had personal control (internal control) over the regretted activity, it was associated with active attempts to change the regrettable behavior, and hence reduced regret as well as reduced rumination. In contrast, for older adults, internal attributions were instead associated with more intense regrets. Research have shown that older adults differ from younger adults in the use of regret regulatory strategies they use to regulate their daily decision regret, this also indicates that lower negative emotions in older adults is not a consequence of declining cognitive ability but rather a consequence of older adults using emotional regulatory strategies (Bjälkebring et al., 2013).

Finally, we want to stress the importance of validating the age differences found in cross-sectional experimental studies such as our own with longitudinal studies. There are several longitudinal studies already that could possibly verify at least some of the cross-sectional findings in the literature (H70, OCTO TWIN, HRS, BETULA, Seattle Longitudinal Study, the Baltimore Longitudinal Study of Aging, etc.). In cases where longitudinal studies cannot verify the cross-sectional findings such longitudinal studies should be conducted.

Research has only scratched the surface of understanding age related changes in cognition, affect, and motivation. This provides both challenge and an opportunity for researchers to fill the gaps. The next few decades will be very interesting. We can only hope to, as the Vulcans say, “dif-tor heh smusma” or in English “live long and prosper.”

## Author contributions

PB drafted the manuscript, and DV, SD, and PS provided critical revisions. All authors approved the final version of the manuscript for submission.

## Funding

The first author is funded by The Swedish Research Council Award Number 2016-00507. This research was supported by grant from the Swedish Research Council for Health, Working Life and Welfare (FORTE, previously FAS) and Centre for Aging and health—AGECAP.

### Conflict of interest statement

The authors declare that the research was conducted in the absence of any commercial or financial relationships that could be construed as a potential conflict of interest.
